# Incision of the Sphincter Vaginæ in Certain Cases

**Published:** 1885-07

**Authors:** W. A. Morris

**Affiliations:** Austin, Texas


### INCISION OF THE SPHINCTER VAGINÆ IN CERTAIN CASES

*By W. A. Morris, M. D., Austin, Texas.*

Read before Travis County Medical Society, and published by resolution of Society. For Daniel's Texas Medical Journal.

For many years I have thought of the propriety of presenting to the profession some views in reference to difficult labor, particularly in prima para cases, wherein a rigid unrelaxed sphincter seemed the whole and only resisting force. Where the perinæum has been distended to the utmost power of resistance without laceration and alone kept in that condition by the rigid sphincter. If any one will examine carefully in such cases he will certainly find with every contraction of the womb a strong string-like band surrounding the external os which must either yield to a long continued force, or laceration of the perinæum results.

We often hear of a rigid perinæum, even when the perinæum has yielded to the utmost capacity.

I have recently had an obstetrical case presenting some serious features, which has mainly induced me to lay my views before the profession.

I have been engaged in the practice of medicine over fifty years. About forty-eight years ago I was called up hurriedly,at night, to visit a lady near by who had been in labor nearly twenty-four hours, primapara, and who was in charge of a midwife. As was then the custom,I found the lady sitting on the lap of a friend, the perinæum being supported by the midwife. Upon examination I found the head almost freed from the pelvic cavity and incased, as it were, in the distended perinæum and rectum.

The external os was not more than one and a half inches in diameter. While having the perinæum supported I at once corded the arm and opened a vein. The loss of blood produced no change. So self-evident was it to me that the child could not be born without extensive laceration, unless relief was promptly given, I determined to incise the sphincter vaginæ, which was done by inserting a director between the fœtal head and the distended integuments. I made a lateral and downward incision so as to avoid the perineal body and rectum in the event of further laceration. As soon as it was done, the babe was born. I scarcely had time to receive it securely,so rapidly did it escape, and so small was the injury to the mother that it did not delay her one day in her recovery.

The wound contracted and healed kindly, without any other interference than cleanliness and protection.

The operation, undoubtedly, saved her from an extensive laceration, and probably saved the life of the child.

I have reviewed my course frequently in regard to the operation, and I have never once doubted the propriety of my action. I firmly believe that if the operation was performed more frequently, that it would often prevent much suffering on the part of the mother, and save the lives of many children, which otherwise would be sacrificed by delay.

After waiting for hours with distended perinæum, with but little dilatation and unyielding sphincter, our patient becoming exhausted, the womb vigorously contracting, both the laceration of perinæum and death of the fœtus justly anticipated,shall we wait till one or the other happens as is often the case? If the former, a painful operation, must be performed with doubtful success; if the latter, a life is sacrificed, and the anticipations of the parents blighted. Who does not feel the responsibility in such trying moments? And after every duty has been discharged with skill and sound judgment, odium will often rest upon the devoted head of the accoucheur.

What danger from the operation to the mother? Not so much as from the forceps. If you divide an artery, you have it under control, it leaves no bad consequence. No entailment of trouble of any kind. It does not inflict as much pain as one effort at expulsion. When you divide the sphincter vaginæ the tension is relieved, for that is the resisting force, relaxation at once ensues, and if further laceration takes place it does not involve the perineal body or rectum, and the floor of the pelvis is preserved. You perform a safe and simple operation in order to prevent the dire train of consequences that may, and often do, follow.

The time when to operate must depend upon the judgment of the accoucheur, as it does in every other important operation.

Playfair says "when the tension of the perinæum is so great that laceration seems inevitable, it is generally recommended that a slight incision be made on each side of the central raphe with the view of preventing spontaneous laceration." This may no doubt be done with perfect safety, "but," he adds, "I question if it is likely to be of any use."

The above plan of operating is objectionable for the reason,if further laceration takes place, it is in the direction of the rectum, and must pass through the perineal body. If you incise the sphincter vaginæ at the proper angle you avoid the mischief. Are we justifiable in performing the operation? I am aware of the danger of recommending such a procedure. I know that many objections will be urged against it. I can anticipate them. It might be abused, and the operation be performed in many cases when not necessary, and where the natural efforts would accomplish the object in view. Admitted; but who would discard the most important operations of the present day because of the want of good, practical sense in the operator?

Incision of the os uteri is a recognized operation where other methods have failed to dilate. In such cases neither the forceps nor ergot is available. Under the same circumstances we should divide the sphincter vaginæ, for if we do not operate, we can do nothing but await the final result, and that too often ends in laceration of the perinæum, or death of the child.

Lacerations are not of uncommon occurrence, as every practitioner is aware.

Simpson says "evidence of the great frequency of laceration of the anterior structure of the perinæum is furnished by almost every careful autopsy of women after delivery, whether assisted or not assisted during their labor. These lesions are not, as has been alledged, necessarily the result of mismanagement, but they occur constantly in practice, despite every modification of management, and in cases also in which no kind of management has been adopted."

Duncan: "Surely now-a-days most patients and nurses know that it is now unavoidable."

Numerous cases are reported in our medical journals, as well as recorded in our text books, wherein the operation might have been justifiably performed.

In the May number of the Courier Record, Dr. S. F. Starley reports a case of a young married lady who, after having been in labor forty hours, was delivered by the forceps, which resulted in the laceration of the perinæum down to the anus. This case certainly would have justified the operation. Nothing was said as regards the fate of the child.

The consequences resulting from protracted labor to the child is also of great importance. Immediate death under the most skillful management often takes place. As the following case from Meigs exemplifies: "I once attended a young woman in labor with first child. The process was most tedious. The head was fully six hours pressing on the perinæum and external parts, under violent uterine contraction. The child was at length born, but was dead." If the child escape immediate death, convulsions often ensue, ending in death, as I have witnessed, or an impairment of the full developement of the brain and its functions may follow.

To the mother also, the consequences of protracted labor are very grave. In the February number of the Courier Record, Dr. Starley, of Corsicana, uses the following forcible language:

"No more truthful maxim was ever uttered than that of the illustrious J. Y. Simpson 'that the mortality of child birth is in direct proportion to the duration of labor.' Physiology teaches that prolonged muscular effort loads the blood with effete material, renders the system feverish, and tends to the developement of local engorgements, inflammations and suppurations. Nothing can be more certain than that the nervous exhaustion and depressed vitality consequent upon a protracted and difficult labor is one of the most frequent causes of disease in the puerperal state. During the stage, when the womb does powerfully contract, and in close contact with the infant, when the placental circulation therefore is, or may be, partially interfered with, and when the soft parts of the mother, both the uterus and other parts below are necessarily subjected to great pressure, the results of labor become far more serious, swelling, œdema, inflammation, with subsequent loughing, and fistula occur. The child may die from continued compression of its skull, cord or placenta, and general symptoms of exhaustion and collapse take place, from which the woman, if not promptly delivered, may die on the spot, or succumb afterwards, from post partem hemorrhage, puerperal inflammation, septicæmia, etc. I make one more quotation, and that from Dr. Thomas, setting forth the consequences to the mother: "As for me, I fully confess that at the moment of labor, I would rather a patient sustain a fracture of the radius, than a laceration of the perinæum down to the sphincter ani. The broken bone would cause pain, sleeplessness, nervousness and perhaps fever, but it would not expose the patient to the same danger of septicæmia or of subsequent uterine, vaginal, rectal or vesical displacements. Let us suppose that the perinæum has been torn during labor down to the sphincter ani muscle. In this accident the vagina is always torn, and an immediate consequence is,the exposure of an extensive raw surface. Over this surface the flow of an ichorous, fetid and semi-putrid animal fluid must, in spite of the greatest precautions, steadily pass for from two to three weeks, a fluid consisting of decaying and flaking decidua, disorganized blood and quantities of muco-pus. The wonder is not that septicæmia occurs so often under the circumstances, but that so many escape it, when everything seems perfectly arranged to favor it."

I will be pardoned for making the above lengthy quotations as they bear such importance to my subject.

In conclusion, if I am correct in my position, and our ablest obstetricians are not mistaken in regard to the dangers incident to prolonged labors, then we are justified in dividing the sphincter. Now, I therefore, after mature deliberation, submit this to the profession for their indorsement or censure. I ask that it be weighed in the balance, and if found wanting, that it be consigned to the great ocean of forgetfulness, where all unworthy productions should go; if anything worthy to live, whereby human suffering may be ameliorated,and dangers averted by judicious and timely interference in that class of cases, which are paramount to all others, wherein the welfare and lives of two beings are jeopardized, the mother and her offspring, then let it live.

